# Relation between Selenium and Female Fertility: A Systematic Review

**DOI:** 10.1055/s-0042-1744288

**Published:** 2022-06-03

**Authors:** Luiz Gustavo Lima, André Amaro Mamédio dos Santos, Tiago Daniel Gueiber, Ricardo Zanetti Gomes, Camila Marinelli Martins, Andrielle Cristina Chaikoski

**Affiliations:** 1 Department of Medicine, Universidade Estadual de Ponta Grossa, Ponta Grossa, PR, Brazil

**Keywords:** selenium, fertility agents, female, reproduction, selênio, fármacos para a fertilidade feminina, reprodução

## Abstract

**Objective** To analyze the influence of selenium in female fertility.

**Data source** A search was performed in the following databases: MEDLINE, Web of Science, Scopus, SciELO, LILACS, MDPI, ScienceDirect, and Europe PMC. The descriptors selected were “ *selenium* ” AND “ *female* ” AND “ *fertility* ”. The search interval was from 1996 to 2021.

**Study selection** The evaluation was performed independently by two reviewers, and a third reviewer confirmed the inclusion of papers in case of divergence between the first two reviewers. Papers were selected after the title and abstract were read, and those that met the eligibility criteria had the full text read.

**Data collection** The following data was extracted: author, year of publication, country, type of study, objective, method, sample size, follow-up period, patients' mean age, inclusion and exclusion criteria, and concentration of serum and capillary selenium. The data was organized in chronological order of paper publication.

**Data synthesis** The number of papers identified totaled 3,800, out of which 7 were included in the systematic review. The studies indicated a positive correlation between serum selenium and antioxidant concentration in the follicular fluid, reduction in antithyroid antibodies, oocyte production and follicle number.

**Conclusion** Selenium supplementation is promising in women with this micronutrient deficiency to promote improvement of the reproductive efficiency and prevent damage to the pregnancy. Further studies on this theme are still required.

## Resumo

**Objetivo** Analisar a influência do selênio na fertilidade feminina.

**Fonte dos dados** Uma busca foi realizada nas seguintes bases de dados: MEDLINE, Web of Science, Scopus, SciELO, LILACS, MDPI, ScienceDirect e Europe PMC. Os descritores selecionados foram “ *selenium* ” AND “ *female* ” AND “ *fertility* ”. O intervalo de busca foi de 1996 a 2021.

**Seleção dos estudos** A avaliação ocorreu de maneira independente por dois revisores, sendo que um terceiro corroborou a eleição dos artigos em casos de divergência. Os estudos foram selecionados através da leitura do título e resumo, e aqueles que contemplaram os critérios de elegibilidade foram lidos na íntegra.

**Coleta dos dados** Os seguintes dados foram extraídos: autor, ano de publicação, país, tipo de estudo, objetivo, método, tamanho da amostra, tempo de acompanhamento, média de idade das pacientes, critérios de inclusão e exclusão, concentração de selênio sérico e capilar. Os dados foram organizados em ordem cronológica de publicação do estudo.

**Síntese dos dados** Foram identificados 3.800 artigos e incluídos 7 estudos na revisão sistemática. Os resultados indicaram correlação positiva entre o nível de selênio sérico e a concentração de antioxidantes no fluido folicular; diminuição dos níveis de anticorpos antitireoidianos; produção de oócitos, e número de folículos.

**Conclusão** A suplementação de selênio é promissora em mulheres com deficiência do micronutriente, a fim de promover melhora na eficiência reprodutiva e prevenir danos na gravidez. Salientou-se a necessidade de realização de mais estudos sobre o tema.

## Introduction

Selenium (Se) is an important micronutrient for several vital functions, such as deoxyribonucleic acid (DNA) synthesis and modulation of the thyroid metabolism, 1 in addition to promoting the regulation of antioxidant levels in the organism through its action in the glutathione peroxidase (GPx) active center. 2 This mineral is found in both organic (around 90–95%) and inorganic forms. 3 Its organic presentation in the human body occurs as selenomethionine and selenocysteine, while its inorganic presence is observed as selenate or selenite associated to other micronutrients. 4 Food items that are rich in selenium include seafood, meat, cereals, grains, and dairy products. 5

Some divergence is found in the literature regarding the recommended values of selenium for human ingestion, and the Institute of Medicine (EUA) recommends 55 μg/day for adult women, and 70 μg/day for adult men. 6 The maximum daily amount tolerable for ingestion is 400 μg/day. 7 As for the Se status evaluation in human beings, the measurement of its plasma concentration is considered the most efficient method, which enables the analysis of short-term variations, upon the nutrient ingestion. 8 The mean Se serum concentration among countries is diverse; for example, in Finland the mean concentration is 41.7 μg/L, while in Brazil it is 73.2 μg/L, and in Canada 158.3 μg/L. 9 10 In addition, studies have revealed that the prevalence of serum hyposelemia is high in different populations worldwide, mainly in Europe and Asia. 11 Global estimates point to half a billion individuals with deficiency of this mineral. 11

Recent studies have reported that Se deficiency might also result in pregnancy complications, such as spontaneous miscarriage, damage to the fetus nervous and immunological systems, in addition to being a risk factor for low weight at birth. 2 Several selenoproteins are found in mammals, such as GPxs, iodothyronine deiodinases (DIOs), thyroxines (Trxs), and thyroxine reductases (TrxRs), 12 while the selenoprotein P (SELENOP) and the iodothyronine deiodinase 3 (Dio3) are expressed in the uterus. 13 Selenoproteins play an important role in many biological functions, such as antioxidant defense and the formation of thyroid hormones. 14

Nevertheless, the oxidative stress characterized by an increase in the oxygen reactive species (ORS) and/or decrease in antioxidant systems is a well-established cause for the senescence of somatic cells, 15 in addition to being one of the potential causes of gamete aging. 16 One research has already elucidated that serum selenium and selenoprotein levels are increased in large healthy follicles and might play a vital antioxidant role throughout later growth and the proliferation of follicles. 17

Some studies reported that higher ORS and inflammatory biomarker levels play a central role in the development of intrauterine pathologies. 18 Moreover, when reproductive organs suffer strong oxidative stress, a failure in the hormone synthesis occurs, which might result in infertility. 14 Also, oxidative stress interferes in the embryo implantation and the pregnancy development; Se increases the migration and proliferation of trophoblast that forms the external layer of the blastocyst, reducing the mitochondrial oxidative stress. 19

As for Se's role in fertility, several studies pointed out that this nutrient has a fundamental role in male reproduction, being essential for normal testicle development, spermatogenesis, and sperm motility, 20 mainly through the selenoproteins GPx and SELENOP. 21 In a double-blind randomized placebo-controlled trial in which there was supplementation of antioxidants, including Se, an increase in the rate of viable pregnancy compared with the placebo group was observed in patients undergoing intracytoplasmic sperm injection (ICSI). 22 Thus, selenium supplementation with doses below 200 μg/day is likely to be beneficial to men regarding sperm motility improvement, 19 which has been confirmed by findings in the literature in which men supplemented with this daily dose associated with vitamin E showed increased sperm motility, morphology, and spontaneous pregnancy rate. 23

Regarding female fertility, the relation between Se status and the Se-dependent GPx activity was demonstrated in a few studies, which observed that relatively low serum and follicular levels are associated with a higher infertility occurrence. 24 Selenium deficiency was associated with ovarian degeneration and follicle atresia in rats. 25 On the other hand, higher Se ingestion through diet was associated with reduction in the growth of primordial, secondary, and antral follicles, stroma, and blood vasculature in the ovary of sheep fetuses when compared with normal levels of Se ingestion. 26

Lack of accuracy and insufficient data hamper the recommendation of routine measurement of Se concentration regarding its role in male and female reproduction seeking to solve fertility problems. 19 For this reason, the present study aimed to carry out a systematic review on the impact of Se on female fertility.

## Methods

This study is registered at PROSPERO under the number CRD42021244985. The report was written according to the recommendations of the Preferred Reporting Items for Systematic Reviews and Meta-Analysis (PRISMA).

This systematic review sought to answer the following question: “What is the relation between Se and female fertility?” The inclusion criteria were studies performed on women in different age groups whose main aim was to analyze the influence of Se supplementation on female fertility. The search was intended to elucidate whether the presence of this mineral interferes in women's sexual physiology. The exclusion criteria adopted were studies performed on animals or male individuals, research involving joint supplementation with other micronutrients, literature reviews, systematic reviews, and metanalyses.

The search was performed on eight databases, namely, MEDLINE, Web of Science, Scopus, SciELO, LILACS, MDPI, ScienceDirect, and PMC. The paper could be in any language, but most of the papers found were in English; the search period was from 1996 to 2021. The last search date was 02/23/2021. The descriptors used in all bases were *selenium* AND *female* AND *fertility* .

The study selection was performed independently by two reviewers using the Mendeley platform (Mendeley Ltd., London, LND, UK). Initially, the papers were selected through the reading of title and abstract, and those that satisfied the eligibility criteria and were chosen by both reviewers had the full text read for inclusion or exclusion in the review. A third reviewer helped to confirm the selection of papers that generated divergence between the first two reviewers.

The studies included had their data summarized by two evaluators in a Microsoft Excel (Microsoft Corp., Redmond, WA, USA) spreadsheet, in which the following items were identified: author, year of publication, country, type of study, study objective and method, sample size, follow-up time, patients' mean age, body mass index (BMI), inclusion and exclusion criteria, measurement methods, supplementation, serum Se concentration, and capillary Se concentration.

The characteristics of the samples in each study were analyzed, and the objectives and results were presented to verify whether Se caused interference in fertility. With that purpose, a chart was built with the following characteristics of each study: author/year of publication, country, type of study, sample mean age, sample size, objective, and methodological quality.

The evaluation was performed by two independent reviewers. The methodological quality of the observational studies was measured employing the Newcastle-Ottawa scale. In that scale, the scores of the studies were calculated based on 8 items, grouped into 3 components: selection (0–4 points), comparability (0–2 points), and outcome (0–3 points). Each item could score a point, except for the ‘comparability item’, which could score 2 points. Studies that scored 9 points were considered of high quality; 6 to 8 points represented moderate quality; those that scored 5 or less points were classified as low-quality. 27 The clinical tests were evaluated using the Physiotherapy Evidence Database (PEDro) quality scale. This scale presents 11 evaluation items that, except for the first, were ascribed 1 point to each item contemplated, totaling 10 points. The following scoring bands in the PEDro scale were adopted: 6 to 10 points = high quality; 4 to 5 points = moderate quality; and 0 to 3 points = low quality. 28

## Results

**Fig. 1:**
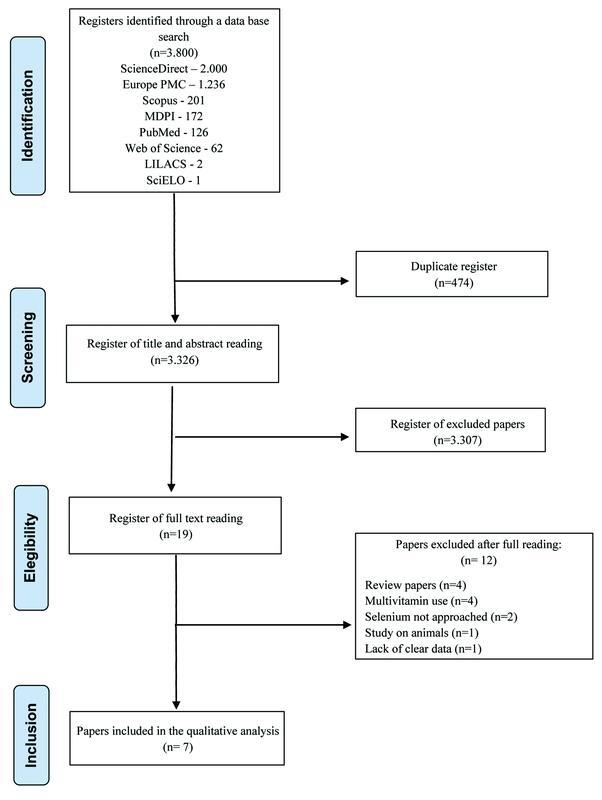
Flowchart of all registers and the selection process performed in each phase of the systematic review on selenium and female fertility.

Out of the 3,800 papers found, 126 were found on MEDLINE, 62 on Web of Science, 201 on Scopus, 1 on SciELO, 2 on LILACS, 172 on MDPI, 2,000 on Science Direct, and 1,236 on PMC. All papers were attached to the Mendeley software for later exclusion ( Fig. 1 ). Four hundred and seventy-four duplicated papers were excluded and 3,326 remained for the primary analysis. In the selection of title and abstract, 3,307 papers were excluded, and 19 remained for full reading. After full reading, another 12 studies were excluded from the review for the following reasons: review papers (4), simultaneous use of other nutrients (4), not approaching Se effect (2), studies on animals (1), and lack of clear data (1). Therefore, this review analyzed 7 papers.

Seven papers were included in this review; among them, five were prospective cohorts, 29 30 31 32 33 one was a case-control, 34 and one was a randomized placebo controlled double-blind clinical trial. 35 The studies were developed in different countries, such as the United States, 29 United Kingdom, 30 India, 31 Poland, 32 Spain, 33 South Africa, 34 and Italy. 35 The publication period was from 2011 to 2020, and the patients' mean age ranged between 29.6 and 32.75 years old. Only one study 29 did not inform the patients' mean age; however, their age range was informed. Regarding the sample size, great difference was noticed, since the smallest included 30 women, 30 while the largest reported 1,249 female participants. 33 All publications included are listed in Chart 1 .

**Chart 1:** Study on selenium and female fertility included in the systematic review and evaluation of methodological quality

Author/Year of publication	Country	Type of study	Mean age (years)	Sample size	Study aim	Methodological quality
Kim et al. (2018) 29	United States	Prospective cohort	18–44 ^1^	259	To investigate the association between 10 minerals and reproduction hormones	Moderate quality *
Dickerson et al. (2011) 30	United Kingdom	Prospective cohort	32.7	30	To assess the exposure to mercury, zinc, and selenium in the gonadal response in IVF	Moderate quality *
Singh et al. (2013) 31	India	Prospective Cohort	32.1	340	To evaluate the relation between oxidative stress and elements of the follicular fluid in women subjected to IVF	High quality *
Ambroziak et al. (2017) 32	Poland	Prospective Cohort	29.6	74	To determine whether selenium is associated to the AITD during pregnancy	High quality *
Lozano et al. (2020) 33	Spain	Prospective Cohort	30.7	1,249	To investigate the association between maternal selenium serum levels and their babies' anthropometry	High quality*
Thomas et al. (2013) 34	South Africa	Case-control	32.75	48	To evaluate whether selenium deficiency is found in women with RPL	Moderate quality *
Mantovani et al. (2019) 35	Italy	Randomized clinical trial	31.9	45	To investigate whether selenium supplementation has a protective effect on thyroid during and after pregnancy	High quality **

Abbreviations: IVF, in vitro fertilization; RPL, recurrent pregnant loss; AITD, autoimmune thyroid disease.

1–Participants' age group;

*Newcastle-Ottawa Scale;

** PEDro scale.

The evaluation using the Newcastle-Ottawa scale pointed out 3 high-quality and 3 moderate-quality studies. The detailed description of the components evaluated is listed in Chart 2 . The PEDro scale evaluation pointed out the high-quality clinical trial ( Chart 3 ). Therefore, we could observe that the studies showed moderate-to-high quality, with low bias risk.

**Chart 2:** Newcastle-Ottawa scale of the cohort and case-control studies included

Study	Design	Selection	Comparability	Outcome	Total
Kim et al. (2018) 29	Prospective cohort	4	1	1	6
Dickerson et al. (2011) 30	Prospective cohort	3	1	3	7
Singh et al. (2013) 31	Prospective cohort	4	2	3	9
Ambroziak et al. (2017) 32	Prospective cohort	4	2	3	9
Lozano et al. (2020) 33	Prospective cohort	4	2	3	9
Thomas et al. (2013) 34	Case-control	4	2	2	8

**Chart 3:** PEDro scale score of the clinical tests included

Study	PEDro scale criteria									
	2	3	4	5	6	7	8	9	10	11
Mantovani et al. (2019) 35	Y	N	Y	N	N	N	Y	Y	Y	Y

Abbreviations: Y, yes; N, no.

(2) - randomized allocation; (3) - blind allocation; (4) - similar groups; (5) - Participants' blinding; (6) - Therapists' blinding; (7) - Blinded evaluators; (8) - proper follow-up (< 15% sample loss); (9) - intention-to-treat analysis; (10) - Comparison between groups; (11) - use of accuracy and variability measures

The objectives and approaches of the studies were different, which made them obtain varied results. The study developed in the United States 29 sought to investigate whether a diet below the daily recommendation of some minerals, including Se, would interfere with the levels of reproduction hormone and increase the risk of anovulation in healthy women. The prospective cohort included 259 healthy women and reported that Se ingestion below the recommended dietary allowance (RDA) was associated with increased risk of sporadic anovulation when compared with the ingestion of higher amounts than those recommended (relative risk [RR]: 2.66, confidence interval [CI] 95%: 0.96;7.36).

A study performed by Dickerson et al. 30 investigated the correlation of capillary Se, zinc, and mercury levels with ovarian hyperstimulation results in in vitro fertilization (IVF). The study included 30 sub fertile women subjected to only one IVF cycle. As for the capillary Se level, significantly positive correlations were found both for oocyte retrieved after ovarian stimulation ( *p* = 0.03, coefficient β = 0.21) and for the number of follicles after ovarian stimulation ( *p* = 0.04, coefficient β = 0.22). It seems relevant to emphasize that the measurement of Se in the hair was chosen for being a reliable result of long-term environmental exposure and dietary status.

The study developed by Singh et al. 31 aimed to evaluate the oxidative stress levels and some oligoelements, Se among them, in the follicular fluid of 140 women with tubal infertility and 200 with endometriosis subjected to IVF. The results of that study showed that Se levels were significantly lower in the endometriosis group than in the tubal infertility group ( *p* < 0.001). In addition, a positive correlation was found between the level of Se and antioxidant concentration (r = 0.63, *p* < 0.001). 31

A study reported by Thomas et al. 34 investigated whether a decline of Se would be found in women with recurrent pregnant loss (RPL). To achieve its aim, two groups of 24 women each (healthy pregnancy and RPL) were compared using capillary Se analysis. The authors observed that no statistically significant difference was observed between the groups regarding Se ingestion, serum Se concentration median ( *p* = 0.219), or in the women's hair ( *p* = 0.74).

The Spanish 33 study evaluated serum Se levels in pregnancy in relation to the gestational age and the newborn's anthropometric data. That study joined two cohorts performed in the municipalities of Valencia and Gipuzkoa, totaling 1,249 mother-child pairs. At the end of the study, a weak insignificant inverse relation was observed between the newborn's cranial circumference and maternal Se concentration ( *p* = 0.08), and a borderline statistically significant direct relation between maternal Se concentration and lower gestational age (hazard ratio [HR]: 1.007, *p* = 0.06).

In Italy, a randomized clinical study 35 evaluated the supplementation of 83 μg/day L-selenomethionine (L-Se-Met), a relevant selenoprotein that has a protective effect on the thyroid during and after pregnancy. The study included 45 pregnant women diagnosed with autoimmune thyroid disease (AITD) who were divided into two groups (supplement and placebo), and it pointed out that Se supplementation provoked decrease in the antithyroid antibody levels after delivery. A significant difference was observed between the supplement and placebo groups regarding the level of thyroglobuline antibody (TgAb) ( *p* = 0.01); thyroid anti-peroxidase antibody (TPOAb) ( *p* = 0.01), and serum Se ( *p* < 0.01). As for the thyroid stimulating hormone (TSH), no significant difference was found ( *p* = 0,09). In addition, the supplement group L-Se-Met showed an increase in the serum Se levels throughout the pregnancy ( *p* < 0.02), an outcome that was opposed to that observed in the placebo group.

The study by Ambroziak et al. 32 investigated whether there were differences regarding the serum Se and SELENOP status between healthy pregnant women and pregnant women who had been diagnosed with AITD in the first third of the pregnancy. The study included 74 women, and, at the beginning of the period, 12 women presented AITD and 17 other patients developed the disorder during pregnancy, resulting in 29 pregnant women with AITD and 45 healthy women. A positive correlation was obtained between serum Se and the SELENOP enzyme in the 3 periods observed (r = 0.55 and *p* < 0.001, in the 1 ^st^ third of the pregnancy; r = 0.34 and *p* < 0.01, in the 2 ^nd^ third; r = 0.51, and *p* < 0.001 in the final third). A significant decrease in serum Se and SELENOP was observed throughout the pregnancy in both groups; however, no significant difference was found between them. Another analysis was that no relation occurred between SELENOP, and TPOAb and TgAb autoantibody levels, or between serum Se and TgAb. However, low correlation was observed between serum Se and TPOAb (r ^2^ = 0.1004, *p* = 0.0042).

Serum Se levels measured during and after pregnancy in the above mentioned studies can be observed in Chart 4 . It was observed that, in the supplemented group, there was no decrease in the Se level during pregnancy, differently from the other groups.

**Chart 4:** Mean serum Se concentration by trimester of pregnancy in the studies

Pregnancy trimester	Mean serum Se concentration (μg/L)	SD
1st trimester		
Healthy pregnant women 32	64.60	14.60
Healthy pregnant women 33	79.57	9.64
AITD pregnant women 32	66.60	12.60
Placebo pregnant women 35	70.26	29.06
Supplemented pregnant women 35	70.09	30.95
2nd trimester		
Healthy pregnant women 32	57.80	14.00
AITD pregnant women 32	59.00	9.90
Placebo pregnant women 35	56.24	19.48
Supplemented pregnant women 35	91.53	25.49
3rd trimester		
Healthy pregnant women 32	48.40	11.30
AITD pregnant women 32	52.20	11.60
6 months postpartum		
Placebo women 35	71.80	18.83
Supplemented women 35	93.54	23.53

Abbreviations: AITD, autoimmune thyroid disease; SD, standard deviation; Se, selenium.

## Discussion

The studies included in this systematic review were mostly prospective cohorts; therefore, it was impossible to reach a conclusive analysis regarding the effect of Se supplementation on women's fertility. Those reports were published from 2011 onwards, and most of them 29 32 33 35 in the last 4 years, indicating scientific interest in the theme.

Similar to study by Kim et al., 29 the case-control study developed by Maeda et al. 36 analyzed the influence of Se and other heavy metals in women's fertility. The study included 98 infertile women that received infertility treatment and 43 healthy women, and it showed that Se plays a protective role in female fertility.

The studies by Dickerson et al. 30 and Singh et al. 31 demonstrated the relevant role of Se in follicles, regarding both quantity (number follicles), and quality (antioxidants). These results confirm the study by Paszkowski et al., 37 who observed low Se concentrations in the follicular fluid of women subjected to IVF or that presented unexplained infertility. In addition, these findings were also evidenced in the literature review reported by Mintziori et al., 19 which suggested that the presence of low Se concentrations in the follicular fluid is a marker for unexplainable infertility, and stated that Se supplementation was beneficial to women subjected to assisted reproductive technologies (ARTs).

Also, regarding the Se antioxidant effect, Ceko et al. 17 highlighted that Se supplementation might alleviate ovarian pathologies resulting from antioxidant expression reduction, such as the selenoprotein GPx1. It seems relevant to mention that a decrease in antioxidants increases ROS formation, which might lead to oocyte development inability. This corroborates the study by Razavi et al., 38 in which Iranian women with polycystic ovarian syndrome (POS), which presents the characteristic of high inflammatory activity, presented higher pregnancy rate following Se supplementation. This result might have occurred due to increased serum Se levels that provided a higher antioxidant capacity, enabling greater success in fertilization.

The results by Mantovani et al. 35 confirm with the findings of the systematic review and metanalysis reported by Zuo et al., 39 which evidenced that Se supplementation for AITD treatment caused great reduction in TPOAb levels (IC 95%: -4.06; -96.43, *p* < 0.00001). Some decrease was also noticed in TgAb levels; however, these were not significant. Conversely, Ambroziak et al. 32 did not find correlation between the serum Se status and the TgAb antibody level. However, a low positive correlation was observed between the serum Se level and TPOAb. The divergence between these studies shows that the role of Se in the thyroid is not well understood, and it might even have a deleterious activity in the gland.

Regarding the study reported by Lozano et al., 33 other similar studies in human beings were not found in the literature. These findings do not agree with those reported by Meyer et al. 40 and Vonnahme et al., 41 who supplemented Se in pregnant sheep to evaluate the maternal and newborn's body performance and composition. The sheep that presented nutritional restriction and were supplemented showed improved fetus growth, which indicates that mineral deficiency might have negative consequences to the gestational development.

The divergences found between the results of the studies on the impact of Se on fertility confirm Gierus's 42 statement about the Se double role as an essential nutrient that can be highly toxic. Thus, we highlight the need for further research to establish the Se-fertility relationship, mainly randomized double-blinded clinical trials. A possible example of this dichotomy is the case-control study developed by Thomas et al., 34 as they analyzed whether low level of capillary Se were involved with RPL. No differences were found between women with RPL and those with healthy pregnancies. This suggests that Se did not present influence regarding pregnancy development in healthy pregnancy or pregnancy loss.

Another type of analysis of the influence of Se on pregnancy refers to its role in pregnancy-induced hypertension. In this sense, in the randomized and placebo controlled double-blinded clinical trial developed by Rayman et al., 21 primiparous pregnant women received 60 μg/day of Se supplementation from the 12 ^th^ gestational week up to delivery. That study included 230 pregnant women, and its primary outcome was obtained upon analysis of the concentration of the receptor-1 of the vascular endothelial growth factor soluble in serum (sF1t-1), an antiangiogenic factor linked to the preeclampsia risk. The sF1t-l concentration was significantly lower in the supplemented group when compared with the placebo group in participants of the lowest quartile of Se status at the beginning of the study ( *p* = 0.039). Therefore, those authors concluded that Se supplementation has the potential of reducing the risk of preeclampsia in pregnant women with Se deficiency.

Also, regarding the influence Se in preeclampsia, a prospective cohort reported by Lewandowska et al. 43 compared healthy primiparous pregnant women who developed pregnancy-induced hypertension with pregnant women who remained normotensive. They found out that the group presenting hypertension showed a reduced level of serum Se when compared with the healthy group ( *p* = 2.6 × 10 ^−10^ ). Therefore, they concluded that Se might be considered a relevant marker for pregnancy-induced hypertension risk.

To sum up, it seems relevant to emphasize that studies regarding the influence of Se on female fertility are still scarce in number and evidence, which makes it impossible to draw solid conclusions on the theme. Qazi et al. 1 reinforced the need to carry out further studies to clarify the role of Se and selenoproteins in the ovarian physiology, fetal development, pregnancy related complications, impact on the placental oxidative stress, and female reproductive success.

**Fig. 2:**
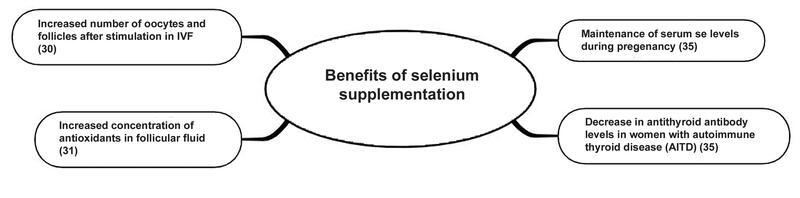
Benefits of selenium supplementation found in this review.

Another fact observed is that none of the studies found evaluated the influence of high levels of Se and toxicity. Thus, Se supplementation only seemed to be promising in women with mineral deficiency, confirming the hypothesis defended by Rayman et al. 21 A summary of the potential benefits of Se supplementation found in this review can be observed in Fig. 2 .

Finally, some limitations of this review were the few studies found investigating Se supplementation, mainly placebo controlled randomized double-blinded clinical trials; few studies evaluating female reproductive success; and absence of analysis of the supplementation impact on older women's fertility. These difficulties reduce the possibility of generalizing the findings for the clinical practice. In addition, the studies included in this review presented varied approaches and methods, which made it impossible to carry out a metanalysis. No other systematic review on this subject was found in the literature.

## Conclusion

The present review pointed out that Se supplementation is promising in women with deficiency of this micronutrient to promote improvement in the reproductive efficiency, mainly in women subjected to IVF. Although few studies on the theme were identified, this review evidenced that the maintenance of normal levels of Se might prevent possible damage to pregnancy, such as preterm birth, preeclampsia, in addition to reducing antibody rates in women with AITD. Another finding was that the antioxidant role of Se might alleviate damage caused by follicular fluid oxidative stress. However, further studies are still needed to elucidate the influence of Se on female fertility and, mainly, the impact of high concentrations of this mineral on women's bodies.
